# Adult Congenital Heart Disease in Serbia: Insights from a Single-Center Registry

**DOI:** 10.3390/diagnostics15040498

**Published:** 2025-02-19

**Authors:** Aleksandra Nikolić, Stefan Veljković, Jovana Lakčević, Ana Peruničić, Armin Šljivo, Miloš Babić, Marko Nikolić, Slobodan Tomić, Dragana Radoičić, Mihajlo Farkić, Darko Boljević, Sanja Vučinić, Sanja Kablar, Milovan Bojić

**Affiliations:** 1 Cardiovascular Institute “Dedinje”, 11040 Belgrade, Serbia; 2 Faculty of Medicine, University of Belgrade, 11000 Belgrade, Serbia; 3 Department of Cardiosurgery, Clinical Center of University of Sarajevo, 71000 Sarajevo, Bosnia and Herzegovina; 4 Faculty of Medicine, University of Banja Luka, 78000 Banja Luka, Bosnia and Herzegovina

**Keywords:** adult congenital heart disease, mortality rates, clinical outcomes, multidisciplinary care

## Abstract

**Background/Objectives:** Congenital heart disease (CHD), affecting approximately 1% of live births, has transitioned to a chronic condition due to advances in diagnostics and surgery, resulting in an increasing adult congenital heart disease (ACHD) population. This study characterizes the clinical and demographic profiles of ACHD patients in Serbia, focusing on congenital anomalies, mortality rates, and key clinical factors to identify opportunities for improving care and outcomes. **Methods:** This observational single-center study was conducted at the Cardiovascular Institute “Dedinje” in Belgrade, Serbia, involving patients diagnosed or treated for CHD between 2006 and 2022. **Results:** A total of 1532 patients were included in the study, with common diagnoses including atrial septal defects (ASD) (47.65%) and ventricular septal defects (VSD) (13.19%). The mean patient age was 48.31 years, with a slight predominance of females (57.21%). The complexity of CHD was categorized as mild (54.6%), moderate (36.5%), and severe (6.3%). The mortality rate was 4.2%, with higher rates observed in conditions like Ebstein anomaly (17.78%) and congenital aortic stenosis (11.76%). **Conclusions:** This study provides a comprehensive overview of the current state of ACHD management in Serbia, highlighting the high prevalence of ASD and VSD among patients, the challenges associated with moderate and severe CHD, and the notable mortality rates for certain conditions. The findings underscore the importance of improving early detection, individualized treatment plans, and multidisciplinary care to enhance patient outcomes in this growing population.

## 1. Introduction

Congenital heart disease (CHD), the most common congenital anomaly affecting approximately 1% of live births globally, has transitioned from a predominantly pediatric condition to a chronic disease of adulthood due to advancements in diagnostic imaging, interventional cardiology, and surgical techniques, resulting in improved survival and a growing population of adults with congenital heart disease (ACHD) who require lifelong specialized multidisciplinary care [1,2,3,4,5].

The rising prevalence of ACHD introduces a complex array of challenges, necessitating advanced multidisciplinary management, longitudinal surveillance, and nuanced risk stratification to mitigate morbidity and mortality [2]. As these individuals age, they often experience new or worsening cardiac issues, including arrhythmias, heart failure, valve dysfunction, and the late sequelae of previous surgical interventions. The heterogeneity of ACHD means that each patient presents with unique needs that evolve over time, necessitating individualized care plans that incorporate various medical specialties, such as cardiology, surgery, obstetrics, and psychology. To address these intricacies, the implementation of ACHD registries has become indispensable, facilitating the systematic acquisition and analysis of detailed patient-specific data, including demographic variables, phenotypic presentations, therapeutic interventions, and prognostic outcomes [2].

However, most registries of ACHD patients have been established in developed countries with a higher number of ACHD centers, whereas data on the ACHD population originating from transitional countries remain scarce. This study aims to characterize the clinical and demographic profiles of patients with ACHD captured in our institutional registry, including the prevalence of specific congenital anomalies, mortality rates, and other pertinent clinical parameters. By elucidating these data, we seek to provide a comprehensive overview of the current state of ACHD management in Serbia and to identify critical areas for targeted interventions aimed at optimizing patient outcomes and advancing care delivery.

## 2. Materials and Methods

This observational single center study was conducted at the Cardiovascular Institute “Dedinje” in Belgrade, Serbia, encompassing patients with CHD who were either diagnosed or treated during childhood or diagnosed in adulthood. The inclusion criteria were as follows: (i) patients with a CHD diagnosis who were referred to, diagnosed, or treated at our center between 2006 and the end of 2022, (ii) patients who provided informed consent for participation in the registry, and (iii) patients aged 18 years and older, although there were couple of patients (3) who were included before age 18, who were referred to our Institute for a specific evaluation and/or intervention. In Serbia, our center is the only registered ACHD center and institution that regularly evaluates and admits patients with ACHD, and currently there is no national database regarding this population. Ethical approval for the study was granted by the Bioethical Committee of the Cardiovascular Institute “Dedinje”, ensuring compliance with ethical guidelines and the principles of the Helsinki Declaration, with particular emphasis on protecting patient rights, privacy, and confidentiality throughout the research process.

### 2.1. Data Collection and Patient Classification

Demographic and clinical data were systematically collected from the medical records and electronic databases of the Cardiovascular Institute “Dedinje”. The collected data encompassed a range of variables, including gender, age at diagnosis, the type of CHD diagnosed, and the patient’s survival status at the time of the study. In addition, detailed clinical information was obtained, such as the presence of comorbidities, the nature of any surgical or interventional treatments received, and the timing of these interventions relative to the patient’s diagnosis.

For patient classification, individuals were categorized based on the type of CHD they presented with, which included conditions such as atrial septal defects (ASD), ventricular septal defects (VSD), tetralogy of Fallot, and other congenital heart malformations. Additional subgroups were created based on the need for surgical or interventional procedures, allowing for the evaluation of long-term outcomes associated with various treatment strategies. This classification enabled a comprehensive analysis of disease progression, treatment effectiveness, and survival rates within the cohort.

Most individuals were followed up; however, long-term follow-up for some patients was not possible due to significant population migration following the war(s). Many individuals and families were displaced either internally or internationally due to socio-economic instability, healthcare system restructuring, and changes in residency status. To address this, we attempted to track these patients through the **Ministry of Internal Affairs**, as well as the **Institute of Public Health of Serbia**, and medical records, but we were unable to obtain sufficient follow-up data.

### 2.2. Statistical Analysis

Descriptive statistics were used to summarize the patient characteristics. Categorical variables were presented as frequencies and percentages, while continuous variables were presented as the mean ± standard deviation or median (interquartile range) as appropriate. All statistical analyses were conducted using SPSS version 26 (IBM Corp., Armonk, NY, USA).

## 3. Results

A total of 2810 patients were identified, out of which 968 patients had diagnosis of a PFO, and 220 had diagnosis of bicuspid aortic valve (BAV); these were not included in the study. Of remaining 1622 patients with CHD that were included and registered in the study, 90 patients were lost to follow-up.

**Figure 1:**
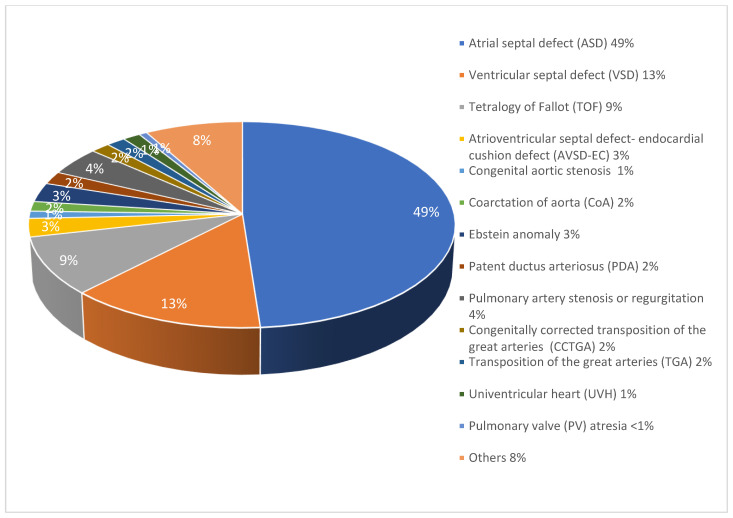
Frequency and percentage of dominant CHD diagnoses in the follow-up cohort.

From the 1532 (94.45%) patients that were followed up, the most common CHD diagnoses were ASD (47.65%), and VSD (13.19%), with a higher percentage of females 876 (57.21%) than males 655 (42.79%). The mean age of the patients was 48.31 years, with a range from 14 to 86 years. The mean follow-up duration was 5 ± 2.3 years, with a minimum follow-up of 2 years and a maximum of 16 years. The distribution of the followed-up patients is presented in Table 1 and Figure 1.

Unfortunately, 90 (5.55%) patients were lost to follow-up, with the highest percentage among ASD (28.89%) and Ebstein anomaly (24.44%) patients. The distribution of the patients lost to follow-up are presented in Table 2.

The distribution of CHD complexity, modeled after the ESC 2020 ESC Guidelines for the Management of Adult Congenital Heart Disease [6] showed that 60.7% of patients had mild, 32.1% had moderate, and 5.6% had severe complexity CHD (Table 3).

The remaining 2.6% of patients were unclassified. According to our data, 502 patients (28.7%) underwent adult operations, 158 patients (9.1%) underwent childhood operations, and 95 patients (5.4%) underwent both childhood and adult operations. Additionally, 638 patients (36.4%) did not undergo any surgery. A total of 328 patients (18.7%) received adult interventions, 29 patients (0.9%) underwent both intervention and surgery in childhood, and 1 patient (0.1%) received both intervention and surgery in adulthood. The average age at death was 58.3 years, and, to the best of our knowledge, all deaths were attributed to cardiovascular causes. The mortality rate among the registered CHD patients was 4.2%, with Ebstein anomaly (17.78%), congenital aortic stenosis (11.76%), and CCTGA (12.50%) having the highest mortality percentages (Table 4).

## 4. Discussion

The objective of this comprehensive single-center registry-based study, conducted at Serbia’s high-volume tertiary cardiovascular center “Dedinje”, was to provide an in-depth analysis of the current state of care for ACHD. It is important to emphasize that the Cardiovascular Institute “Dedinje” has had experience in treating ACHD patients since the very beginnings of cardiac surgery, primarily through individual cases. It was widely believed that this population comprised a relatively small number of patients, which is why a structured approach to their care was not established for a long time. A structured and systematic approach to the treatment of these patients began in 2006, marking the start of data collection as well. By examining the distribution and anatomical complexity of CHD diagnoses within this population, this study aimed to identify critical patterns and trends. Furthermore, it sought to evaluate key clinical outcomes, including mortality rates and patient retention in long-term follow-up care, thereby highlighting potential areas for optimization in the management and treatment strategies for this growing patient cohort.

The study found that the most common ACHD diagnoses were ASD and VSD, with a higher percentage of females than males. Factors which may be related to higher female to male prevalence could be of genetic or environmental influences specific to the population in Serbia. Factors such as maternal health, nutrition, and environmental exposures could contribute to a higher incidence of CHD in female infants. However, it is important to note that the overall prevalence of CHD is typically higher in males; so, any observed difference in the ratio of male to female births with CHD could reflect complex interactions between genetics, developmental factors, and regional health patterns. The distribution of CHD complexity showed that 60.7% of patients had mild complexity CHD, 32.1% had moderate complexity CHD, and 5.6% had severe complexity CHD. This distribution aligns with previous research, which has also highlighted that the majority of ACHD patients tend to fall into the mild-to-moderate categories, while a smaller proportion present with severe and more complex conditions. The predominance of mild CHD reflects improved survival rates and long-term management strategies, allowing individuals with less severe defects to reach adulthood and receive specialized care [7,8,9]. The mortality rate among the registered CHD patients was 4.62%, with patients with Ebstein anomaly, congenital aortic stenosis, and CCTGA having the highest mortality percentages. Patients with severe CHD often face significant hemodynamic challenges, such as severe tricuspid regurgitation in Ebstein anomaly or systemic ventricular dysfunction in CCTGA. These conditions frequently require multiple surgeries and carry a substantial risk of complications such as heart failure, arrhythmias, and pulmonary hypertension, contributing to a disproportionately higher mortality rate among these patients [7,8,9].

The high prevalence of ASD diagnoses in this study is consistent with previous studies reporting a high prevalence of these diagnoses in ACHD patients [10,11,12]. This can be attributed to the typically mild or asymptomatic nature of ASDs in early life, which often allows individuals to reach adulthood before detection or the need for intervention. Advances in diagnostic techniques, such as echocardiography, have further contributed to the identification of these defects, even in asymptomatic patients, emphasizing their prominence in ACHD registries. The mortality rates observed in patients with Ebstein anomaly, patients with congenital aortic stenosis, and CCTGA remain relatively high, are concerning, and warrant further investigation. For example, Ebstein anomaly is frequently associated with severe tricuspid regurgitation, right-sided heart failure, and atrial arrhythmias, all of which significantly impact long-term survival. Similarly, congenital aortic stenosis may lead to left ventricular hypertrophy, valvular dysfunction, and heart failure, while CCTGA places the morphologic right ventricle in a systemic position, leading to systemic ventricular failure over time [7,8,9]. These conditions often require repeated surgical or catheter-based interventions, which carry their own risks and long-term implications [13]. These findings underscore the critical importance of ongoing monitoring and risk stratification in ACHD patients, particularly those with more complex CHD. Comprehensive multidisciplinary care involving cardiologists, cardiac surgeons, and other specialists is essential to optimize outcomes for these patients [14]. Risk stratification tools that incorporate clinical, imaging, and biomarker data can help identify patients at higher risk for adverse outcomes, enabling timely interventions and tailored management strategies. Moreover, further research is needed to refine surgical techniques, improve long-term medical therapies, and develop innovative approaches to address the unique challenges associated with complex CHD.

The study also found that 4.94% of patients were lost to follow-up, with the highest percentage among ASD and Ebstein anomaly patients. The disproportionately high percentage of lost-to-follow-up patients in these groups underscores the challenges of maintaining consistent care for ACHD patients. Contributing factors may include a lack of awareness about the need for lifelong monitoring, transitions from pediatric to adult care, geographical barriers to accessing specialized ACHD centers, and socioeconomic factors [15,16,17]. Inadequate communication during care transitions, especially when patients move from pediatric to adult healthcare systems, has been identified as a key contributor to follow-up attrition. These findings highlight the urgent need to implement and enhance strategies to improve follow-up and monitoring for ACHD patients. Potential interventions include establishing robust care transition programs, leveraging telemedicine to reach geographically distant patients, and enhancing patient education about the importance of lifelong follow-up. Additionally, developing centralized ACHD registries could facilitate the tracking of patients and improve care coordination. Tailored risk stratification protocols may help prioritize resources for high-risk patients, such as those with Ebstein anomaly or other complex CHDs, to prevent poor outcomes [18]. ACHD patients with prosthetic valves face a heightened risk of infective endocarditis (IE) due to prosthetic material, altered hemodynamics, and residual shunts, resulting in high morbidity and mortality. Diagnosis is complicated by complex anatomy and prostheses, reducing the sensitivity of standard imaging. Transesophageal echocardiography (TEE) and advanced imaging techniques like 18F-FDG PET/CT provide improved diagnostic accuracy. Given these complications, regular follow-up is essential to detect and manage potential infections early [19].

The Serbian ACHD registry, when compared to established international registries, reveals both strengths and areas for development. The CONCOR registry [20,21] in the Netherlands, initiated in 2001, has enrolled over 16,000 patients across 100 hospitals, facilitating extensive multicenter research that has significantly contributed to the development of ACHD guidelines and international collaborative studies. Similarly, the JNCVD-ACHD [22] registry in Japan, founded in 2011, has amassed data from more than 24,000 patients, with a particular focus on complex congenital conditions such as Fontan physiology and systemic right ventricle. While the Serbian registry addresses similar conditions, it lacks the expansive multicenter collaboration and comprehensive dataset that characterizes these international registries. The NRCHD [23,24] in Germany, established in 2003, provides a further point of comparison. It integrates data from patients of all ages, employing standardized IPCCC coding for phenotypic analysis, which enables precise tracking of diverse congenital heart defects. The registry also includes a biorepository for genetic and molecular research, supporting more advanced investigative endeavors. Its longitudinal tracking of patient outcomes, coupled with its emphasis on collaborative, multicenter studies, positions it as a gold standard for ACHD registries. A notable strength of the Serbian registry lies in its adoption of the ESC classification system, which enhances the accuracy and consistency of CHD diagnoses, thereby allowing for more reliable data analysis and facilitating direct comparisons with international datasets.

The Swedish National Registry for Congenital Heart Disease (SWEDCON), established in 2009, integrates data from pediatric and adult CHD patients, tracking outcomes from fetal stages to adulthood. It includes four sub-registries: fetal, pediatric, congenital cardiac surgery, and GUCH (Grown-Up Congenital Heart). Sweden sees around 1000 CHD births annually, with over 500 pediatric cardiac surgeries performed each year. The adult CHD population exceeds 40,000, particularly those with complex defects [25].

One limitation of this study is its single-center design, which may limit the generalizability of the findings to other settings. Nevertheless, to our knowledge, this study is the first registry-based study of adult congenital heart disease patients in Serbia. The registry-based approach is essential to improve the management and care of ACHD patients, as it allows for the collection and analysis of data related to patient demographics, clinical characteristics, management strategies, and outcomes. By identifying the most common diagnoses, the study provides a better understanding of the current landscape of ACHD care in Serbia. The study also highlights the need for ongoing monitoring and risk stratification in ACHD patients, particularly those with more complex CHD. Further, the high mortality rates observed in patients with Ebstein anomaly, congenital aortic stenosis, and CCTGA warrant further investigation and underscore the need for specialized and comprehensive care for ACHD patients.

## 5. Conclusions

This single-center registry-based study provides important insights into the landscape of adult congenital heart disease care in Serbia. One of the main objectives of the study is to contribute to large global databases with data from transitional countries, such as the former Yugoslav states, providing an insight into the ACHD population in these regions. The study highlights the need for specialized and comprehensive care for adult patients with ACHD, as well as ongoing monitoring and risk stratification. The high prevalence of ASD diagnoses, along with the mortality rates observed in patients with Ebstein anomaly, congenital aortic stenosis, and CCTGA, underscore the importance of ongoing research and investigation into these areas. This study’s results can serve as a valuable resource for clinicians and policymakers working to improve the care and outcomes of patients with adult congenital heart disease in Serbia and other regions.

## Figures and Tables

**Table 1:** Frequency and percentage of dominant CHD diagnoses in the follow-up cohort.

Dominant CHD Diagnosis	Number (*N* = 1532)	Percentage (%)
Atrial septal defect (ASD)	730	47.65%
Atrioventricular septal defect-endocardial cushion defect (AVSD-EC)	43	2.81%
Congenital aortic stenosis	17	1.11%
Coarctation of aorta (CoA)	53	3.46%
Ebstein anomaly	45	2.94%
Patent ductus arteriosus (PDA)	30	1.96%
Pulmonary artery stenosis or regurgitation	67	4.37%
Congenitally corrected transposition of the great arteries (CCTGA)	24	1.57%
Transposition of the great arteries (TGA)	24	1.57%
Tetralogy of Fallot (TOF)	137	8.94%
Univentricular heart (UVH)	22	1.44%
Pulmonary valve (PV) atresia	10	0.65%
Ventricular septal defect (VSD)	208	13.19%
Others	122	7.96%

**Table 2:** Frequency and percentage of dominant CHD diagnoses in the lost-to-follow-up cohort.

Dominant CHD Diagnosis	Number *N* = 90	Percentage (%)	Gender (Male/Female) (30/60)
Atrial septal defect (ASD)	26	28.89%	7/19
Ebstein anomaly	22	24.44%	10/12
Ventricular septal defect (VSD)	15	16.67%	5/10
Tetralogy of Fallot (TOF)	5	5.56%	0/5
Coarctation of aorta (CoA)	4	4.44%	2/2
Pulmonary artery stenosis or regurgitation	4	4.44%	1/3
Patent ductus arteriosus (PDA)	3	3.33%	0/3
Congenitally corrected transposition of the great arteries (CCTGA)	2	2.22%	2/0
Atrioventricular septal defect–endocardial cushion defect (AVSD-EC)	1	1.11%	0/1
Congenital aortic stenosis	1	1.11%	1/0
Univentricular heart (UVH)	1	1.11%	0/1
Others	6	6.67%	2/4

**Table 3:** CHD complexity.

CHD Complexity	Total Number (*N* = 1532)	Percentage (%)
Mild	836	54.6%
Moderate	559	36.5%
Severe	98	6.3%
Unclassified	39	2.6%

**Table 4:** Mortality distribution by CHD type.

CHD Group	Total Patients *N* = 1532	Mortality *N* = 64	Alive *N* = 1468	Mortality Percentage (4.2%)
Atrial septal defect (ASD)	730	20	710	2.74%
Atrioventricular septal defect-endocardial cushion defect (AVSD-EC)	43	0	43	0.00%
Congenital aortic stenosis	17	2	15	11.76%
Coarctation of aorta (CoA)	53	3	50	5.66%
Ebstein anomaly	45	8	37	17.77%
Patent ductus arteriosus (PDA)	30	1	29	3.33%
Pulmonary artery stenosis or regurgitation	67	5	62	7.46%
Congenitally corrected transposition of the great arteries (CCTGA)	24	3	21	12.50%
Transposition of the great arteries (TGA)	24	1	23	4.17%
Tetralogy of Fallot (TOF)	137	12	125	8.76%
Univentricular heart (UVH)	22	1	21	4.55%
Pulmonary valve (PV) atresia	10	0	10	0.00%
Ventricular septal defect (VSD)	208	5	203	2.40%
Others	122	3	119	2.46%

## Data Availability

The data are available upon request.
